# A Comparison of Transcriptional Diversity of Swine Macrophages Infected With TgHB1 Strain of *Toxoplasma gondii* Isolated in China

**DOI:** 10.3389/fcimb.2020.526876

**Published:** 2020-09-25

**Authors:** Yongle Song, Lindong Song, Xiaoting Wan, Bang Shen, Rui Fang, Min Hu, Junlong Zhao, Yanqin Zhou

**Affiliations:** ^1^Key Laboratory Preventive Veterinary of Hubei Province, College of Veterinary Medicine, Huazhong Agricultural University, Wuhan, China; ^2^State Key Laboratory of Agricultural Microbiology, Huazhong Agricultural University, Wuhan, China

**Keywords:** *Toxoplasma gondii*, RNA-seq, pig macrophage, host-parasite interaction, immunity

## Abstract

*Toxoplasma gondii* is an apicomplexan parasite infecting human and animals, causing huge public health concerns and economic losses. Swine alveolar macrophage plays an important role in controlling *T. gondii* infection. However, the mechanism by which macrophages infected with *T. gondii* function in the immunity to the infection is unclear, especially for local isolates such as TgHB1 isolated in China. RNA-seq as a valuable tool was applied to simultaneously analyze transcriptional changes of pig alveolar macrophages infected with TgRH (typeI), TgME49 (typeII) or TgHB1 at different time points post infection (6, 12, and 24 h). Paired-end clean reads were aligned to the Sscrofa10.2 pig genome and *T. gondii* ME49 genome. The differentially expressed genes of macrophages and *T. gondii* were enriched through Gene Ontology and Kyoto Encyclopedia of Genes and Genomes, respectively. Compared to the TgRH and TgME49 infection groups, 307 down-regulated macrophage genes (mainly enriched for development and metabolism) and 419 up-regulated genes (mainly enriched for immune pathways) were uniquely expressed in the TgHB1 infection group. Additionally, 557 down-regulated and 674 up-regulated *T. gondii* genes (mainly enriched in metabolism and biosynthesis) were uniquely expressed in the TgHB1 infection group. For validation purposes, some of the differentially expressed genes of macrophages involved in immune-related signaling pathways were used for further analysis via real time quantitative reverse-transcription polymerase-chain reaction (qRT-PCR). This work provides important insights into the temporal immune responses of swine alveolar macrophages to infection by the strain TgHB1 isolated from China, and is helpful for better understanding of the *T. gondii* genotype-associated activation of macrophages during early phase of the infection.

## Introduction

*Toxoplasma gondii*, as an apicomplexan single-cell parasite, is found throughout the world and causes the zoonotic disease toxoplasmosis. All warm-blooded animals can be infected with *T. gondii* and cats are the definitive hosts where sexual phase of the parasite can be completed. Pigs can be infected with *T. gondii* in the environment via ingesting oocysts discharged by infected cats. Thirty to fifty percent of global population have been exposed to and may be chronically infected with *T. gondii* which has co-evolved with the human population for centuries (McLeod et al., 2009; Flegr et al., 2014). Individuals with weakened immune systems such as the AIDS have the higher risk of developing toxoplasmosis after *T. gondii* infection. Humans and other warm-blooded mammals in many countries can be infected with foodborne pathogen *T. gondii* when they consume raw or undercooked meat such as pork and raw hams containing *T. gondii* tissue cysts or tachyzoites (Dubey et al., 2005; Jones and Dubey, 2012; Sakikawa et al., 2012; de Berardinis et al., 2017; Herrero et al., 2017). Pigs serve as important intermediate hosts between human and *T. gondii*. Much scientific progress has been achieved toward understanding *T. gondii* and toxoplasmosis through experimental studies *in vitro* using human foreskin fibroblast, human embryonic kidney epithelial 293-T cell, mouse RAW 264.7 macrophage cell etc., and in animal models (mouse, feline, pig, etc.). Nevertheless, it is still unclear how *T. gondii* (especially the local isolates) can escape host immunity and survive in human and domestic animals, particularly in their macrophages.

Macrophages formed through the differentiation of monocytes are a type of important cell of the immune system, that are known to protect the host against external dangers, such as pathogens, which can be cleared by these professional phagocytic cells. Macrophages play an essential role in the early immune response against *T. gondii* and are also a predominant *Toxoplasma* infected cell type (Jensen et al., 2011). Through transcriptional analysis of murine macrophages infected with 29 different *Toxoplasma* strains, large differences between strains in the expression level of known parasite effectors, large chromosomal structural variation in some strains, and strain-specifically regulated host pathways have been identified (Melo et al., 2013). *Toxoplasma gondii* replicating in immune cells, such as chicken macrophages and dendritic cells, can serve as a potential source of tachyzoites (Malkwitz et al., 2013; Quéré et al., 2013). Macrophages can ingest the parasite but, in some circumstances, fail to eliminate the sporozoites and tachyzoites (Dubey et al., 1997, 1998; Speer and Dubey, 1998; Zhao et al., 2014; Walwyn et al., 2015), but mouse RAW macrophages are able to facilitate the excystation and differentiation of sporozoites into tachyzoites following oocyst internalization (Freppel et al., 2016). On the other hand, macrophages can also help fight *T. gondii* infection through a purinergic signaling pathway (Petit-Jentreau et al., 2017). For example, human THP-1 macrophages infected with *T. gondii* can produce IL-1β and inflammasome with up-regulated expressions of NOD-Like Receptor related genes (Chu et al., 2016). *Toxoplasma gondii*-infected Lewis rat macrophages can undergo a form of cell death called pyroptosis in order to prevent parasite replication, which may be the reason for Lewis rat's resistance to *Toxoplasma* but the molecular mechanism remains not yet fully understood (Wang et al., 2019).

It has been shown that *T. gondii* dense granule antigen 7 (GRA7) can interact with the cellular TRAF6 factor in the MyD88 pathway, and in doing so, can activate innate immune response in macrophages to provide effective protection against *T. gondii* infection *in vivo* (Yang et al., 2016). It has also been shown that IFN-γ activates macrophages in the classical way and therefore can effectively protect cells against intracellular pathogens (Andrade et al., 2005). Mouse peritoneal macrophages primed with recombinant IFN-β or IFN-γ can inhibit *T. gondii* (type II PLK strain) growth (Mahmoud et al., 2015). However, the mechanism(s) by which macrophages infected with *T. gondii* function in mediating an effective immunity against the infection is unclear. Studying the transcriptomic profiles of pig macrophages infected with *T. gondii* tachyzoites (especially the local isolates) will help understand the mechanism of *T. gondii*–macrophage interactions and will provide new and important insights for the development of novel anti-toxoplasmosis strategies.

TgRH is a type I strain of *T. gondii* with the most virulent phenotype and TgME49 is a type II strain that is less virulent (Saeij et al., 2006; Sibley and Ajioka, 2008; Khan et al., 2009). TgHB1 is a non-canonical type I *Toxoplasma* strain isolated from central China with high virulent phenotype (Zhang et al., 2016). Little is known about how TgHB1 interacts with the host immune cells, especially macrophages. RNA-seq as a powerful tool can be applied to determine gene expression profiles that occur during different experimental conditions. To identify pig macrophage cell signaling pathways uniquely modulated by different *T. gondii* strains, especially TgHB1, pig alveolar macrophages (3D4-21) infected with three different *T. gondii* strains (TgRH, TgME49 and TgHB1) in multiplication of infection 5 (MOI = 5). RNA-seq was employed to obtain the mixed gene expression profiles of the tachyzoites and the infected macrophages at three time points post infection (6, 12, and 24 h) and then used to compare the differentially expressed genes of macrophages infected with TgHB1 relative to other macrophage infected groups in order to reveal unique gene expression/function pathways in pig's macrophage against TgHB1 infection.

## Results

### General Features of the Transcriptomic Data

**Figure 1:**
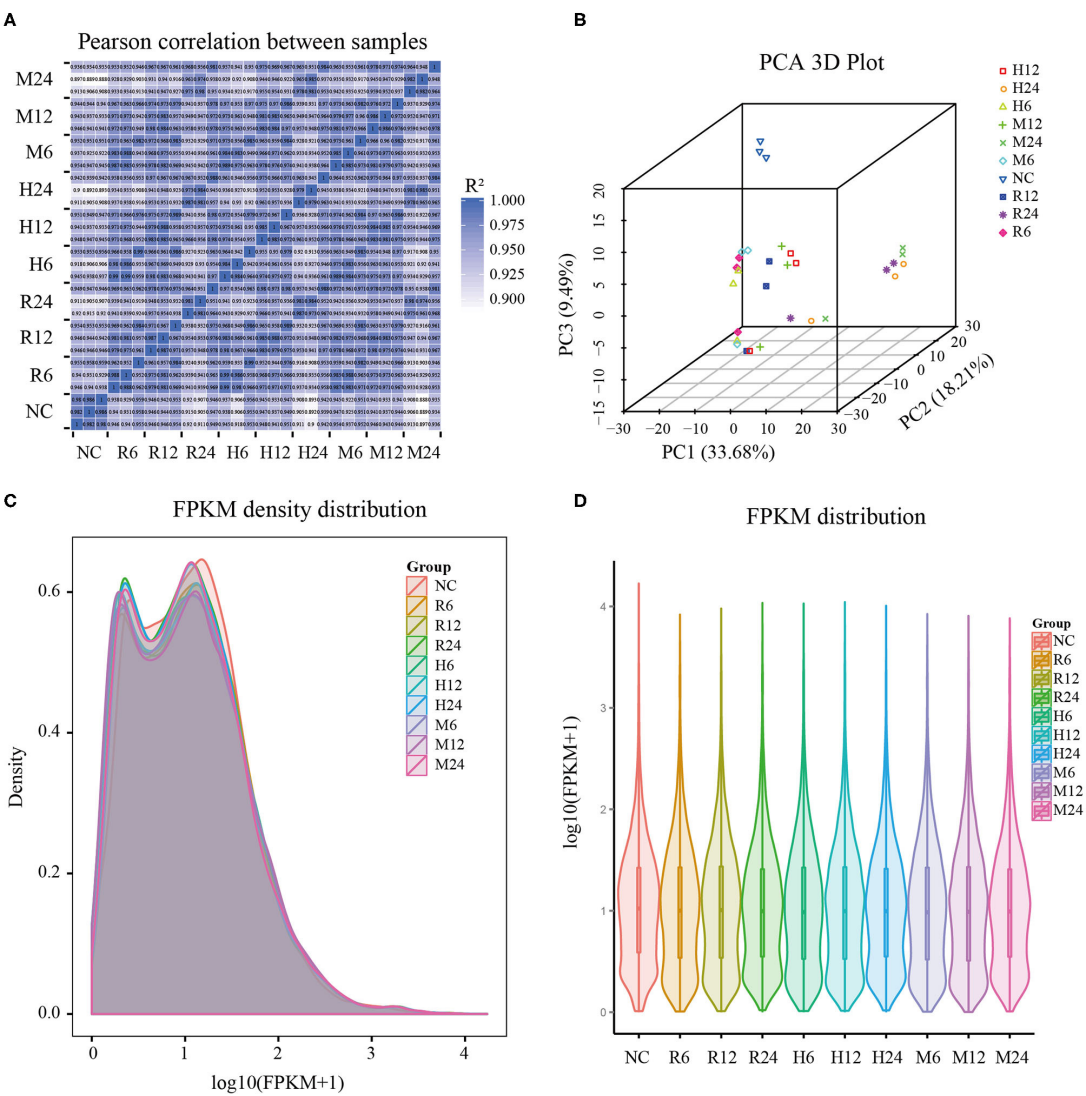
General features of the pig macrophage transcriptomic data. **(A)** Pearson's correlation coefficient analysis; **(B)** Principal component analysis; **(C,D)** The density and violin graph of FPKM distribution. NC represented the control group. R6-24, H6-24, and M6-24 represented the experiment group of pig macrophage infected by RH, HB1, and ME49 strains at 6, 12, and 24 h, respectively.

Total RNAs of the mixture of different tachyzoites (TgRH, TgME49, and TgHB1) and macrophage samples from three time points (6, 12, and 24 h post infection), each with three biological replicates, were collected for RNA-sequencing. We also, respectively, sequenced the control group of uninfected pig macrophages and three different *T. gondii* strains with three biological replicates. The samples of all groups meet the requirement for sequencing through quality inspection by the Agilent 2100 Bioanalyzer. After quality control analysis and removing low-quality reads, over 9G clean bases/sample, including over 84 million clean reads were obtained. These clean reads were uniquely mapped to the reference genome of pig Sscrofa10.2 (GCA_000003025.4) and TgME49 (ToxoDB-41) that have the richest level of annotations for the convenience of subsequent analysis. Most clean reads in all samples (clean reads mapped to host cell and mapped to *T. gondii* in different groups) were distributed into the exon regions, with fewer in the intergenic regions and the intron regions (Supplementary Table 1). Pearson's correlation coefficient among the different time points was over 0.9, which indicated that the correlation between the three biological replicates used in the experiment was satisfactory and the sample data were stable and consistent, and furthermore the gene expression patterns among the samples had high similarity (Figure 1A). The principal component analysis (PCA) showed wide similarity in the host transcriptome among three different strains (Figure 1B).

FPKM (fragments per kilobase per million mapped fragments) was used to normalize the expression level of gene (Trapnell et al., 2010). The density and violin graph of FPKM distribution all showed that the differences of gene expression levels were not significant among the different groups (Figures 1C,D). However, most macrophage genes were in low expression levels after *T. gondii* infection. Uninfected macrophage control group had more genes with FPKM > 1 than 3 different *T. gondii* infection groups at different time points (Supplementary Table 2). So, infection by these different *T. gondii* strains may have an effect on pig macrophage gene expression on the whole.

### Markedly Dissimilar Macrophage Transcriptomes of 3 Different Strains of *T. gondii* at 3 Different Time Points

**Figure 2:**
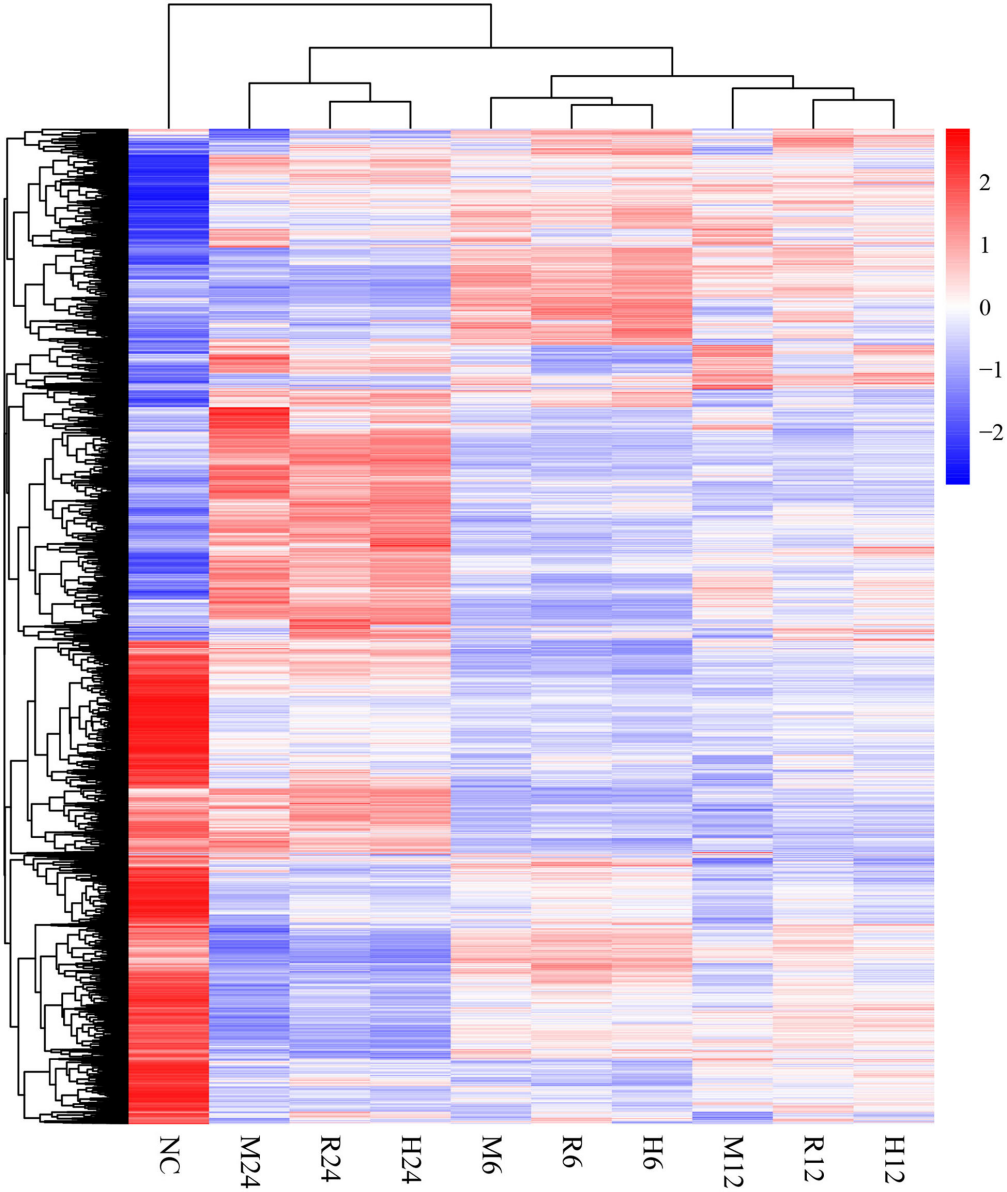
The cluster heatmap of differentially expressed genes of macrophages. NC represented the control group. R6-24, H6-24, and M6-24 represented the experiment group of pig macrophage infected by RH, HB1, and ME49 strains at 6, 12, and 24 h, respectively.

Differential gene expression analysis was performed by comparing the macrophage gene transcriptional level after *T. gondii* infection to that in the control (uninfected) group at each time point using DESeq (padj < 0.05 and | log2 (fold change) | > 1) (Anders and Huber, 2010). The DEseq analysis with a default parameter revealed thousands of pig's and *T. gondii'*s differentially expressed genes among three different strains at 6, 12, and 24 h as compared to the control group, respectively (Tables 1, 2). The gene expressions varied in numbers and in samples. Macrophages infected with TgME49 had more differentially expressed genes than those infected with TgRH and TgHB1. With time, the number of differentially expressed genes in macrophages gradually increased on the whole except the macrophages infected with TgHB1 strain. The cluster heatmap of differentially expressed genes of macrophages in all strains and all time points revealed that macrophages infected with different strains at the same time point showed a similar expression pattern (Figure 2). The transcriptome of macrophages exposed to different strains at 6 h was closer to those at 12 h, and those at 24 h had significantly different expression patterns from those at other time points. Vertical cluster (samples clustering) also showed that transcriptome of macrophages infected with TgHB1 was closer to TgRH group than TgME49 group. It was important and necessary to investigate the exact difference of macrophages exposed to TgHB1 as compared to those exposed to two other strains (TgRH and TgME49) and the unique macrophage pathways in the TgHB1 infected group.

**Table 1:** The number of differentially expressed macrophage genes at different time points.

**Group**	**Comparison method**	**Up-regulated genes**	**Down-regulated genes**	**Total**
RH	6h/0h	762	1,092	1,854
	12h/0h	843	1,120	1,963
	24h/0h	1,301	1,162	2,463
HB1	6h/0h	919	1,267	2,186
	12h/0h	893	1,172	2,065
	24h/0h	1,411	1,253	2,664
ME49	6h/0h	1,101	1,435	2,536
	12h/0h	1,159	1,640	2,799
	24h/0h	1,679	1,568	3,247

**Table 2:** The number of differentially expressed *T. gondii* genes at different time points.

**Group**	**Comparison method**	**Up-regulated genes**	**Down-regulated genes**	**Total**
RH	6h/0h	793	860	1,653
	12h/0h	685	730	1,415
	24h/0h	554	433	987
HB1	6h/0h	1,099	1,236	2,335
	12h/0h	888	840	1,728
	24h/0h	744	612	1,356
ME49	6h/0h	1,023	1,382	2,405
	12h/0h	876	1,263	2,139
	24h/0h	878	1,286	2,164

### Analysis the Differentially Expressed Genes of Macrophages Infected With Each of the 3 *T. gondii* Strains

**Figure 3:**
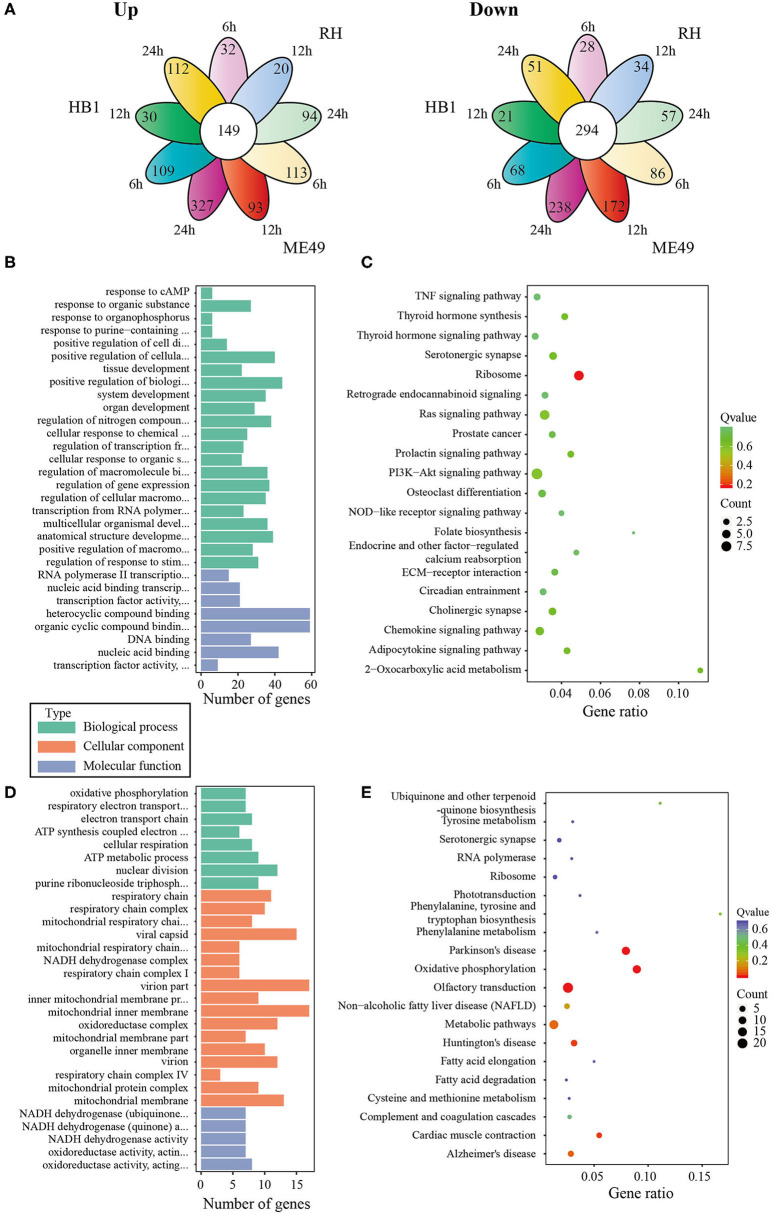
The analysis of differentially co-expressed macrophage genes among three *T. gondii* strains at three time points tested; **(A)** The flower plot of differentially co-expressed macrophage genes; **(B)** The GO term histogram of co-up regulated macrophage genes; **(C)** The KEGG pathway bubble chart of co-upregulated macrophage genes; **(D)** The GO term histogram of co-down regulated macrophage genes; **(E)** The KEGG pathway bubble chart of co-down regulated macrophage genes.

In general, the number of differentially expressed macrophage genes among three *T. gondii* strains at three different time points was ~1,854–3,247 under the different conditions (Table 1). These differentially expressed genes may participate in different pathways and represent different information during macrophage's defense against *T. gondii*. The Gene Ontology (GO) as a convenient tool for the unification of biology was applied to the differentially expressed genes' functional annotation and classification and the Kyoto Encyclopedia of Genes and Genomes (KEGG) was used as a prominent reference knowledge base for integration and interpretation of large-scale molecular data sets to identify the active biological pathways involving *T. gondii* infection (Kanehisa et al., 2008; Young et al., 2010). For complexity among macrophages exposed to three different *T. gondii* strains at different time points, we first concentrated on the differentially expressed genes of macrophages that emerged among all three *T. gondii* strains at three time points. The flower plot showed that 149 co-up and 294 co-down differentially expressed macrophage genes emerged among three *T. gondii* strains (Figure 3A). However, the number of GO terms enriched by co-up differentially expressed genes was many times higher than that enriched by co-down differentially expressed genes (corrected *p* < 0.05). The biological process accounted for the majority GO-term enriched with up-regulated differentially expressed genes among three *T. gondii* strains at three time points, and these 149 co-up differentially expressed genes only involved in two terms related to cellular component (cytosolic ribosome and ribosomal subunit), 12 terms related to molecular function (mainly about transcription factor activity and nucleic acid binding) and 104 term related to biological process (the top three GO term: response to cAMP, organic substance and organophosphorus) (corrected *p* < 0.05). The top 30 GO terms of co-upregulated macrophage genes are showed in Figure 3B. The co-up differentially expressed genes were also enriched into KEGG pathways (the top three pathways: Ribosome, PI3K-Akt signaling pathway and Ras signaling pathway) (Figure 3C). The 294 co-down differentially expressed genes were mainly enriched to cellular component (the top three terms: respiratory chain, respiratory chain complex and mitochondrial respiratory chain), molecular function (mainly about NADH dehydrogenase activity, oxidoreductase activity and cytochrome-c oxidase activity) and biological process (the top three terms: oxidative phosphorylation, respiratory electron transport chain and electron transport chain) (Figure 3D). The co-down differentially expressed genes were enriched into KEGG pathways (the top three pathways: Oxidative phosphorylation, Parkinson's disease and Olfactory transduction) (Figure 3E).

**Figure 4:**
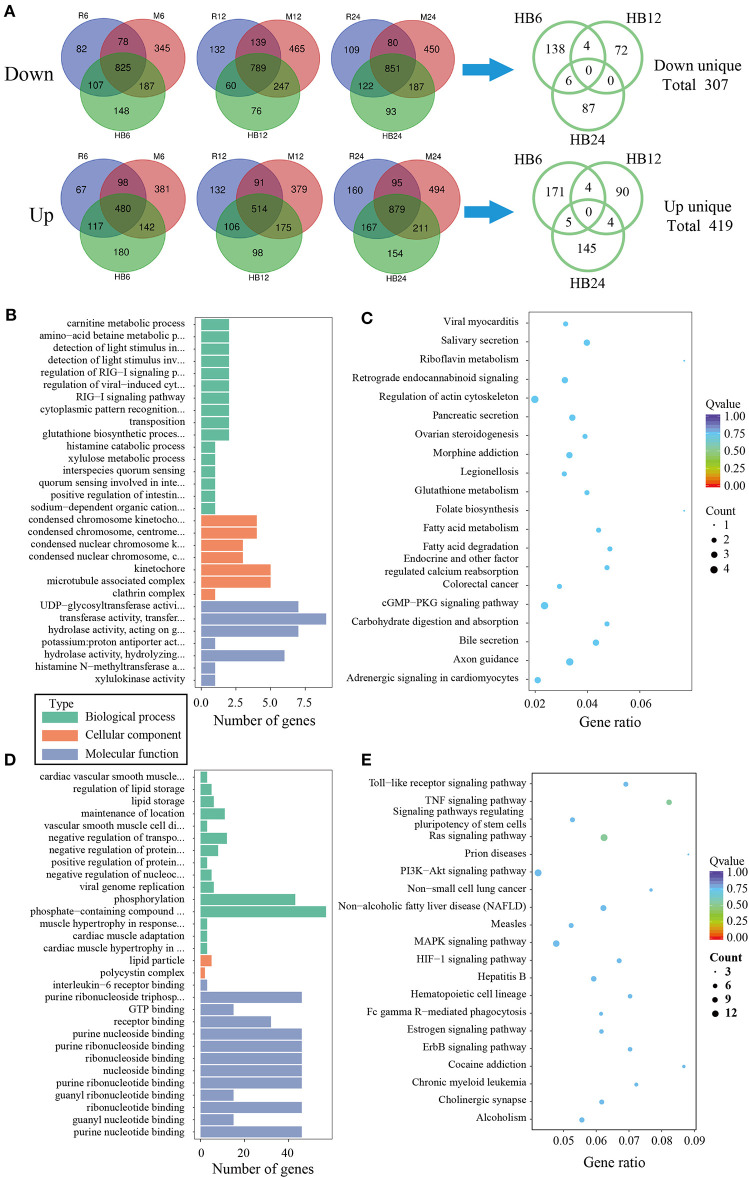
The analysis of unique differentially expressed macrophage genes at TgHB1 group at least at one time point. **(A)** Venn diagram of unique up- and down-regulated differentially expressed macrophage genes in the TgHB1 infection group at least at one time point; **(B,C)** The GO term histogram and KEGG pathway bubble chart of unique down-regulated differentially expressed macrophage genes by the TgHB1 infection group; **(D,E)** The GO term histogram and KEGG pathway bubble chart of unique up-regulated differentially expressed macrophage genes by the TgHB1 infection group. NC represented the control group. R6-24, H6-24, and M6-24 represented the experiment group of pig macrophage infected by RH, HB1, and ME49 strains at 6, 12, and 24 h, respectively.

To analyze the difference of macrophages infected with TgHB1 as compared to TgRH (standard I) and TgME49 (standard II), a Venn diagram was used to depict the unique differentially expressed genes of macrophages infected with TgHB1 at least at one time point (Figure 4A). GO and KEGG analyses revealed these unique genes' potential function and enriched pathways (Figures 4B–E), and those of all uniquely up-regulated macrophage differentially expressed genes (419 genes) from three different time points were mainly enriched in some immune pathways (the top three immune-related pathways: TNF signaling pathway, Ras signaling pathway and Toll-like receptor signaling pathway) but those of the down regulated unique genes (307 genes) were mainly enriched in some pathways related to development and metabolism in macrophages.

### Transcriptional Difference of TgHB1 Compared to TgRH and TgME49 *T. gondii* Strains

**Figure 5:**
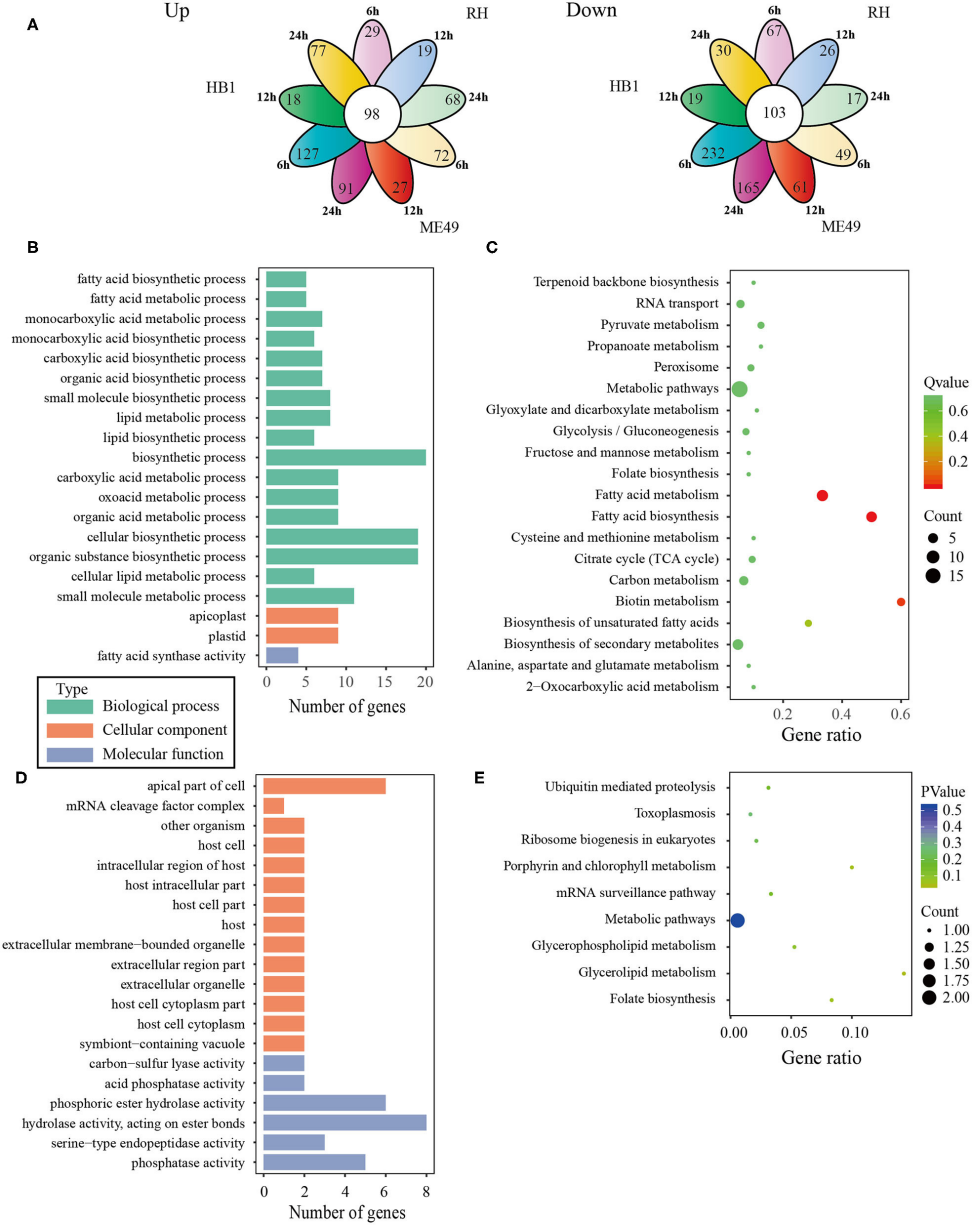
The analysis of differentially co-expressed *T. gondii* genes among three strains at three time points; **(A)** The flower plot of differentially co-expressed *T. gondii* genes; **(B)** The GO term histogram of co-upregulated *T. gondii* genes; **(C)** The KEGG pathway bubble chart of co-upregulated *T. gondii* genes; **(D)** The GO term histogram of co-down regulated *T. gondii* genes; **(E)** The KEGG pathway bubble chart of co-down regulated *T. gondii* genes.

To investigate the difference of three *T. gondii* strains infecting macrophages, the RNA sequencing data of macrophages infected with three *T. gondii* strains among three different time points were aligned to the *T. gondii* TgME49 genome (ToxoDB-41). The differentially expressed genes of three *T. gondii* strains were analyzed in depth. Ninety-eight (98) co-up and 103 co-down differentially expressed *T. gondii* genes emerged among all three strains at three time points (Figure 5A). Almost half of the co-up differentially expressed genes are proteins that possess signal peptide or TM Domains as shown in the ToxoDB (a *Toxoplasma* database) (Supplementary Table 3). The Gene Ontology Enrichment showed that the co-up differentially expressed genes mainly involved in cellular component (plastid and apicoplast), molecular function (fatty acid synthase activity) and biological process (the top three terms: fatty acid biosynthetic process, fatty acid metabolic process and monocarboxylic acid metabolic process). The top 20 GO terms are shown in Figure 5B. The ninety-eight co-up differentially expressed genes were enriched through KEGG (the top three pathways: fatty acid biosynthesis, fatty acid metabolism, and biotin metabolism) and top 20 pathways are shown in Figure 5C. The 103 co-down differentially expressed genes of *T. gondii* included 63 hypothetical proteins with little known information about their functions and 54 differentially expressed genes encoding for proteins that possessed signal peptide or TM Domains (Supplementary Table 3). The targeting and localization of these co-up and co-down regulated *T. gondii* genes were also predicted though another database: Localization of Organelle Proteins by Isotope Tagging (LOPIT) released on ToxoDB website (https://toxodb.org/toxo/). These proteins (total 127 proteins) with the probability threshold of MCMC or MAP above 0.8 were considered credible and the data are shown in Supplementary Table 3. The location at apicoplast includes 22 co-up regulated *T. gondii* genes and those cytosol location related genes (14 *T. gondii* genes) were almost all co-up regulated. However, those dense granules (24 *T. gondii* genes) and microneme (5 *T. gondii* genes) location related genes were mostly down-regulated in all *T. gondii* strains and at different time points. The Gene Ontology Enrichment showed that the co-down differentially expressed genes involved in cellular component (the top three terms: apical part of cell, mRNA cleavage factor complex and other organism) and molecular function (the top three terms: carbon-sulfur lyase activity, acid phosphatase activity and phosphoric ester hydrolase activity). The top 20 GO terms are shown in Figure 5D. The 103 co-down differentially expressed genes enriched KEGG pathways mainly involved in glycerolipid metabolism, porphyrin and chlorophyll metabolism and folate biosynthesis (Figure 5E).

**Figure 6:**
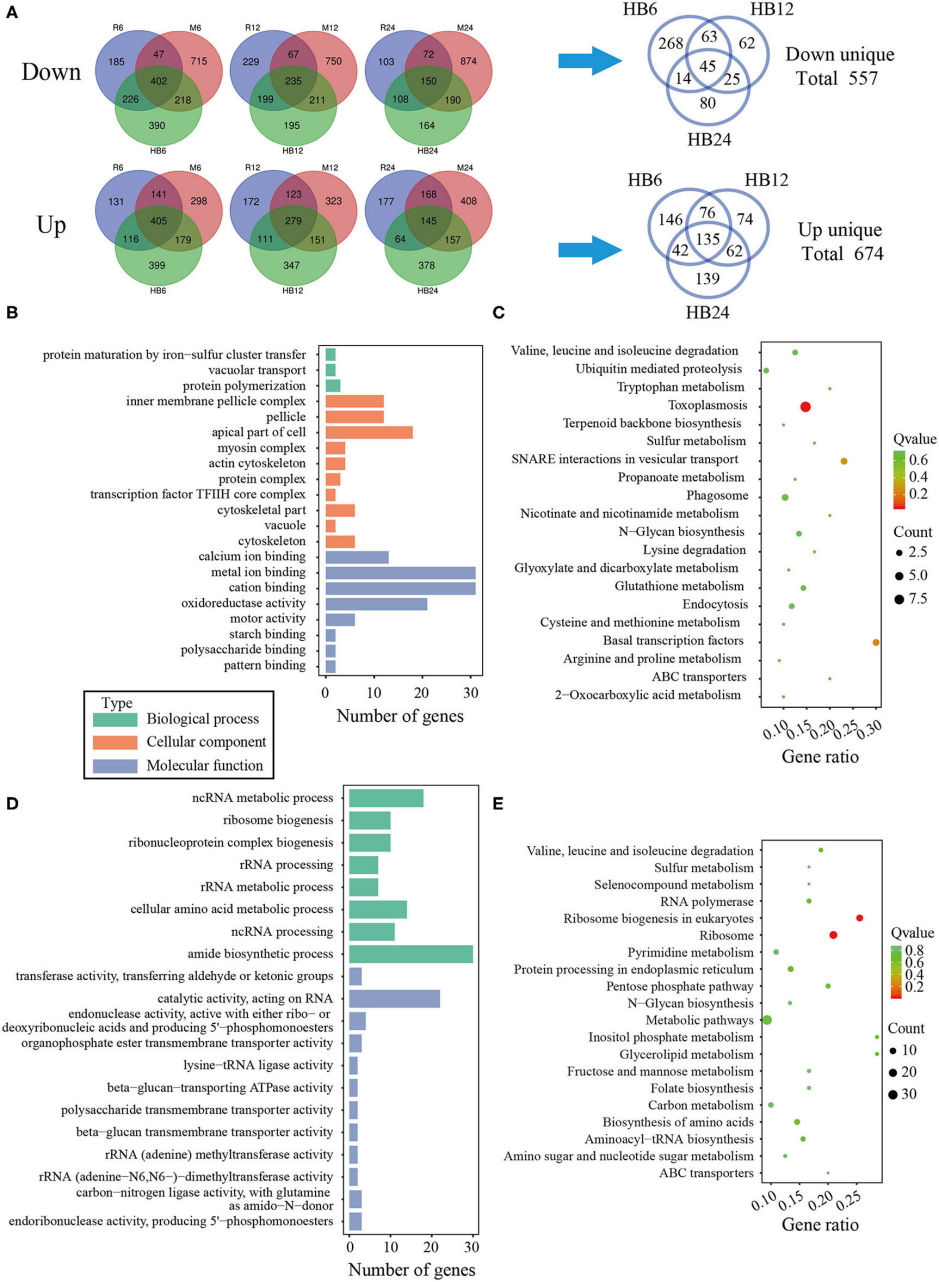
The analysis of unique differentially expressed *T. gondii* genes by the TgHB1 infection group at least at one time point. **(A)** Venn diagram of unique up- and down-regulated differentially expressed *T. gondii* genes by the TgHB1 infection group at least at one time point; **(B,C)** The GO term histogram and KEGG pathway bubble chart of unique down-regulated differentially expressed *T. gondii* genes by the TgHB1 infection group; **(D,E)** The GO term histogram and KEGG pathway bubble chart of unique up-regulated differentially expressed *T. gondii* genes by the TgHB1 infection group. NC represented the control group. R6-24, H6-24, and M6-24 represented the experiment group of pig macrophage infected by RH, HB1, and ME49 strains at 6, 12, and 24 h, respectively.

To reveal the difference of TgHB1 in response of macrophage defense during different time periods of the infection, a Venn diagram was used to depict TgHB1 differentially expressed genes at some time points compared to those of TgRH (standard I) and TgME49 (standard II) (Figure 6A). The Venn diagram of TgHB1 unique differentially expressed genes at three time points revealed 674 co-up regulated genes and 557 co-down regulated genes in TgHB1 at least at one time point. GO and KEGG analysis revealed these genes' functions and enriched pathways. The down-regulated genes were involved in cellular component (the top three terms: pellicle, inner membrane pellicle complex, and apical part of cell), molecular function (the top three terms: calcium ion binding, metal ion binding and cation binding) and biological process (protein maturation by iron-sulfur cluster transfer, vacuolar transport and protein polymerization) (Figure 6B). The down-regulated *T. gondii* unique genes were enriched in KEGG (the top three pathways: toxoplasmosis, basal transcription factors and SNARE interactions in vesicular transport) sorted by corrected p-value then p-value (Figure 6C). However, the up-regulated *T. gondii* genes were involved in molecular function (the top three terms: transferring aldehyde or ketonic groups, catalytic activity acting on RNA and endonuclease activity active with either ribo- or deoxyribonucleic acids and producing 5′-phosphomonoesters) and biological process (the top three terms: ncRNA metabolic process, ribosome biogenesis and ribonucleoprotein complex biogenesis) (Figure 6D). These up-regulated *T. gondii* genes were mainly enriched in KEGG (the top three pathways: ribosome, ribosome biogenesis in eukaryotes and pentose phosphate pathway) (Figure 6E). Localization prediction of these genes is listed in Supplementary Table 4.

### Analysis of Differentially Expressed Macrophage Genes Involved in Immune Pathways

**Figure 7:**
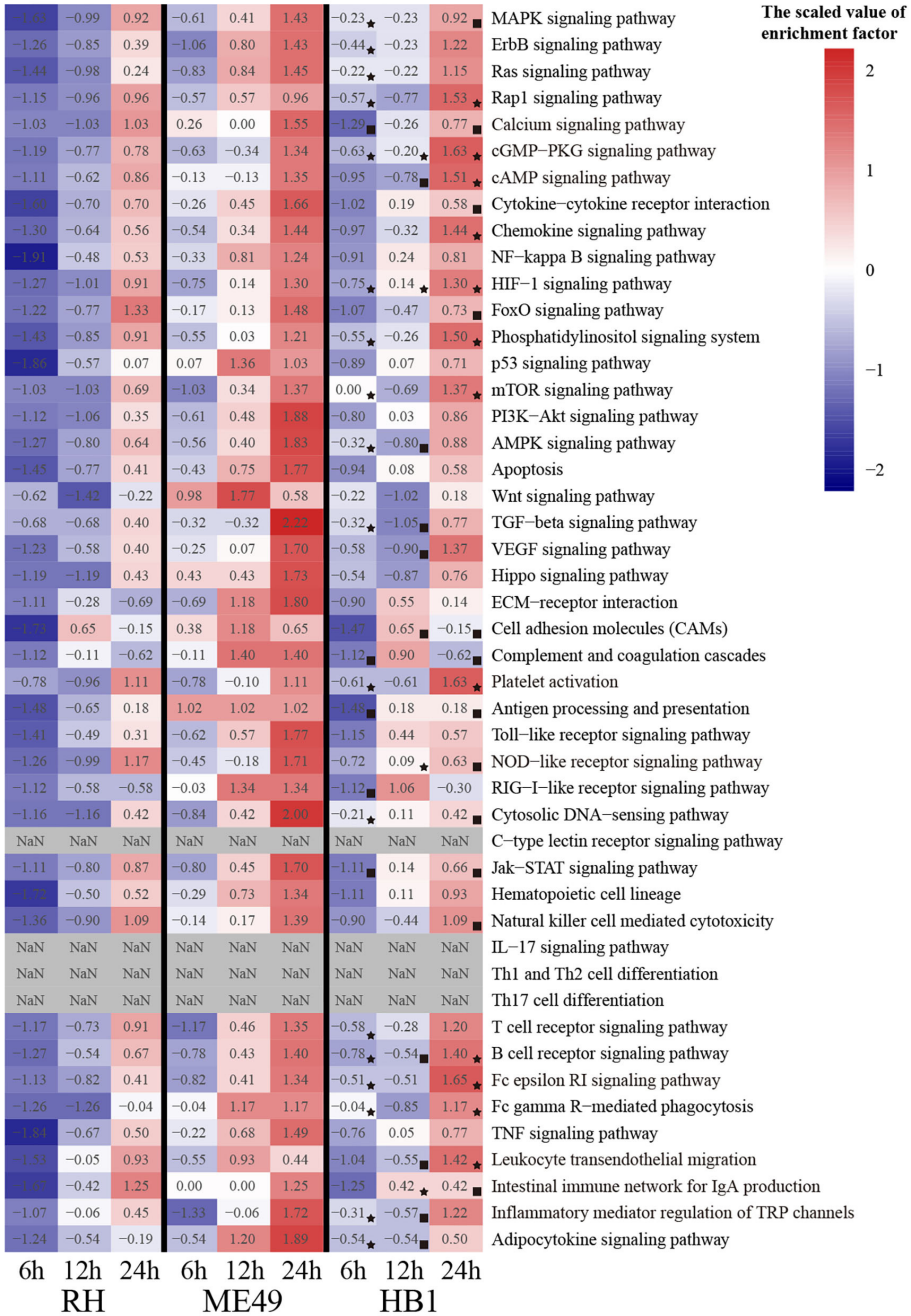
The heatmap of immune-related pathways of macrophages. (⋆) The maximum enrichment factor among the three strains at the same time point. (■) The minimum enrichment factor among the three strains at the same time point.

To compare the immune response related differences of macrophage against TgHB1 China local isolate and the TgRH (standard I) and TgME49 (standard II) strains, we focused on the differentially expressed macrophage genes involved in immune pathways. All differentially expressed macrophage genes were mapped to the KEGG pathways and many pig's immune response related signaling pathways enriched with at least at one time point and in one strain (total 43 KEGG immune pathways mainly about pig immune system and signal transduction), which were summarized for deep analysis (Kanehisa et al., 2017; Cong et al., 2018). The enrichment factor of these 43 KEGG immune pathways was calculated for every *T. gondii* strain and at every time point, then heatmap was applied to show the difference of these immune related KEGG pathways among three *T. gondii* strains and at three time points (Figure 7). Many immune pathways were often enriched within all three *T. gondii* strains at the same time point, though the value of enrichment factor was different. Only four pathways (c-type lectin receptor signaling pathways, IL-17 signaling pathways and Th1 and Th2 cell differentiation and Th17 cell differentiation) were all not enriched in three strains at all time points. These immune related KEGG pathways' enrichment factors of TgHB1 were not synchronized at the same time point comparing to TgRH or TgME49, and their final results may activate or inhibit some immune pathways at different levels. At each time point, the two pathways in TgHB1 infection group (cGMP-PKG pathway and HIF-1 signaling pathway) had higher enrichment factor than the other two strains.

### Validation of Some RNA-Seq Data

**Figure 8:**
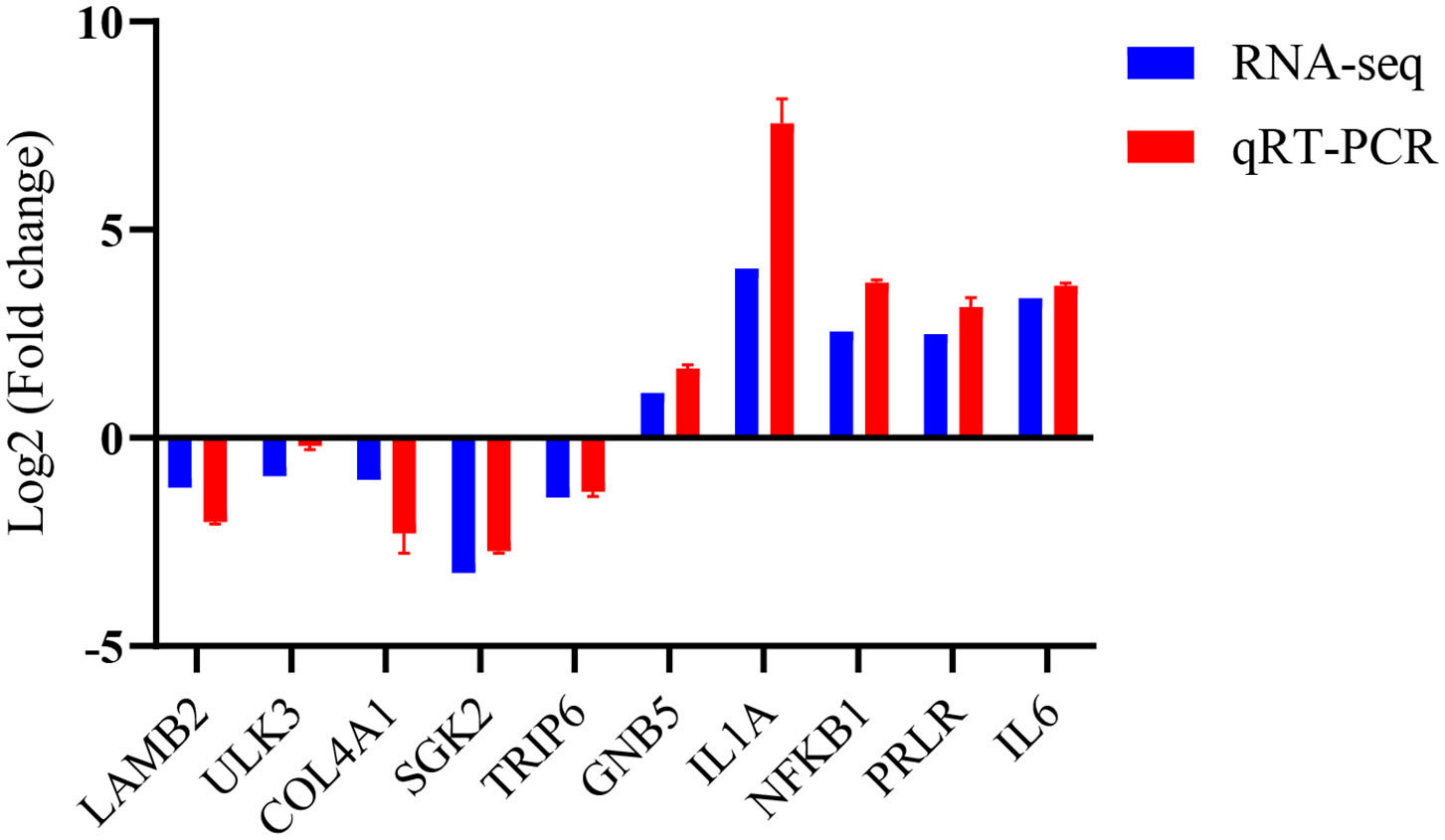
Validation of the RNA-Seq data by real time qRT-PCR.

To verify the accuracy of these RNA data, five up-regulated and five down-regulated representative pig macrophage genes associated with the innate immune pathway were verified through real-time qRT-PCR. The qRT-PCR results of the examined genes at 24 h time point of macrophages infected with TgME49 corresponded well with the RNA-Seq results, confirming the high probability of validity of the RNA sequencing data (Figure 8).

## Discussion

Although foodborne infections are considered the most common routes of toxoplasmosis, the mechanisms of pigs infected with *T. gondii* remain not fully understood, especially the mechanism of clearance of the parasite by the immune system and how parasites survive in immune cells such as macrophages. Transcriptomic studies may provide useful information about the pathogenesis of the infection and host interactions between host cells and *T. gondii*. In recent years, RNA-seq has been widely used to study parasite and host interactions by focusing on gene expression profiling of host cells and/or parasite in order to promote deeper understanding of host and parasite interactions (Wang et al., 2017; Lee et al., 2018). Macrophages play an important role in the host's defense mechanism against *Toxoplasma* infection. Though an increasing number of studies have been published on the host-parasite interaction by using the model organism of *Toxoplasma* and its host cells, investigation into the interaction between *T. gondii* and swine alveolar macrophages seems to be rarely done or insufficient (Zhou et al., 2016). To elucidate the temporal dynamic gene expression patterns of immune cells infected with *T. gondii* and compare the difference between TgHB1 and other known strains, our study focused on comparing the transcriptomic trend of pig alveolar macrophage (3D4/21) infected with two *T. gondii* strains (TgRH, TgME49) and a China isolated strain TgHB1 during a period of time of the infection *in vitro*.

Our study focused on the pattern of changes of macrophage gene expressions after infection by these different *T. gondii* strains and at three different time points (6 h, 12 h, and 24 h). Transcriptomic sequencing produced more than 9GB data/samples in mixed samples that met the requirement for subsequent analysis. As the infection progresses, the number of differentially expressed genes increased slightly on the whole. The final stage (24h/0h) possessed the most differentially expressed genes (Table 1). The number of differentially expressed genes increased with time, which indicated that the macrophages gradually responded to *T. gondii* infection. When comparing TgHB1 with TgRH or TgME49 at three time points, the hierarchical clustering analysis showed that early infection did not induce a significant difference in host cell gene expressions, as three *T. gondii* strains had similar gene expression patterns at the same time point on the whole, but were slightly different in some genes.

When compared the differentially expressed genes of TgHB1 to other known strains (TgRH and TgME49), 149 up-regulated and 294 down-regulated pig macrophage genes expression were realized, which were similar in all three strains and at three time points. These up- and down-regulated genes were only a small part of all differentially expressed genes. It means that most differentially expressed pig macrophage genes were dynamically changing and macrophage infected with TgHB1 was not completely similar to infection by other strains (type I TgRH and type II TgME49). Some over up- or down-regulated genes may activate macrophage to eliminate parasites through IL-12 and IFN-γ, thus controlling parasites infection (Scharton-Kersten et al., 1996; Yap et al., 2000). Next, we focused on identifying the differences in gene expressions between TgHB1 infection and infection by other known *T. gondii* strains. The Venn diagrams showed few TgHB1-infected macrophages differentially expressed genes that were unique and can always be down-regulated or up-regulated only in TgHB1 infection group at all time points tested. Three hundred and seven (307) down- and 419 up-regulated macrophage genes were identified to possess uniquely different transcription pattern in the TgHB1 infection group as compared to the TgRH or TgME49 infection group at least at one time point tested. Three hundred and seven uniquely down-regulated macrophage genes were enriched in secretion and metabolism pathways. However, it was interesting to note that 419 uniquely up-regulated macrophage genes were mainly enriched in immune related pathways (the top three immune related pathways: TNF signaling pathway, Ras signaling pathway and Toll-like receptor signaling pathway). This means that macrophage's immune response to TgHB1 is different than that to the other known *T. gondii* strains (TgRH and TgME49).

To identify the immune-related pathway's difference of macrophages between TgHB1 and others known *T. gondii* strains, we attempted to extract pig immune related KEGG pathways from three different strain-dependent infection groups at three time points. The heatmap analysis of KEGG pathways enrichment factors showed that 43 pig immune related pathways were all enriched with differentially expressed macrophage genes at different levels. The enrichment factors of the same immune related pathways were not completely similar in three *T. gondii* strains at three time points. This means that pig macrophages respond differentially within the same immune pathways at different levels toward the TgHB1 local isolate (atypical) strain. Different *T. gondii* strains might have different adaptability and virulence (Saeij et al., 2005; Sergent et al., 2005). The enrichment factors of cGMP-PKG pathway and HIF-1 signaling pathway in TgHB1 were higher than those of the other two strains. cGMP-PKG signaling pathway is an important pathway for signal transduction which could be activated in porcine alveolar macrophages by carbon monoxide to inhibit porcine reproductive and respiratory syndrome virus replication (Zhang et al., 2017). Hypoxia-inducible factor 1 (HIF-1) is a transcription factor that functions as a master regulator of oxygen homeostasis. HIF-1 signaling pathway is important for the response of macrophage to *Mycobacterium tuberculosis* infection (Elks et al., 2013; Braverman and Stanley, 2017). The high enrichment of cAMP and HIF-1 signaling pathways in TgHB1 may improve the response of pig macrophage to *T. gondii* infection. Our results indicate that pig macrophage responded differentially to a local isolated TgHB1 *T. gondii* strain at some time points tested as compared to other strains (TgRH and TgME49).

To better understand the host-parasite interactions, we also compared the difference in gene expressions of *T. gondii*. After infection, TgHB1 also possessed hundreds of genes differentially expressed at least at one time point as compared to other known *T. gondii* strains. These differentially expressed *T. gondii* genes may be related to the macrophage response differentially toward TgHB1 at three time points tested. However, it was not easy to comprehend the true biological functions for many of these genes as too many hypothetical genes without annotations in the *Toxoplasma* genome were present in the analysis. A total of 1,160 *Toxoplasma* genes were differentially expressed only in the TgHB1 strain at least at one time point (Supplementary Table 4). Many ROP and GRA proteins in the apicomplexan parasites are secreted as effectors that targeted host cells and altered host signaling pathways to favor parasite survival (Hakimi et al., 2017). Protein location prediction revealed that 15 GRA and 28 ROP proteins were only differentially expressed by the TgHB1 strain at least at one time point and most proteins containing SignalP Peptide or TM Domain had the potential to be secreted into host cells and to change macrophage signaling and transcription.

## Conclusion

We performed transcriptomic analysis of pig alveolar macrophages infected with the TgHB1 strain isolated in China at three time points (6, 12, and 24 h) and compared it to the type I and II *T. gondii* strains (TgRH and TgME49). The genes that differentially expressed following *Toxoplasma* infection in pig macrophages may be associated with immune signal pathways and the results may help to better understand the interaction between *Toxoplasma gondii* and its host cells *in vitro* at the transcriptional level. Macrophages infected with TgHB1 were transcriptionally different from those infected with the TgRH or TgME49 strain in different respects, including immune-related pathways. Difference in these immune-related pathways provides important insight into how pig alveolar macrophages response to the local (atypical) strain TgHB1 and our dataset is valuable for deeper understanding of how macrophages resist atypical *Toxoplasma* strain and how the parasite evades immune surveillance. Our study helps to understand the host immunity and defense mechanisms against this parasite in some detail and may help to develop strategies to prevent *T. gondii* infection especially by the local *T. gondii* strain.

## Materials and Methods

### Parasites and Cell Culture

The TgRH (type I) and TgME49 (type II) are known *T. gondii* strains available in our laboratory and TgHB1 is a type I strain isolated from central China that is also available in our laboratory. The confluent monolayers of human foreskin fibroblasts (HFFs) were used to maintain the three *T. gondii* strains in Dulbecco's modified Eagle medium (DMEM) supplemented with 2% fetal bovine serum (Gibco), 2 mM L-glutamine, 100 U/ml penicillin, and 10 ug/ml streptomycin at 37°C in 5% CO_2_ incubator. Pig alveolar macrophages (3D4/21) were used for RNAseq sample collections in RPMI 1,640 medium with 2% fetal bovine serum (Gibco).

### Tachyzoites and Macrophage Samples Collection

Tachyzoites freshly egressed from human foreskin fibroblast monolayers were harvested and filtered through 3.0 um membrane filters (Whatman). Pig alveolar macrophages were infected with tachyzoites of different strains (TgRH, TgME49 and TgHB1) at MOI of 5. The parasites and macrophages were harvested together using 0.05% trypsin digestion at 6, 12, and 24 h time point, respectively. Each experimental group had three biological replicates.

### RNA Extraction and Qualification

Total RNA was extracted individually from mixture samples using Transzol UP Reagent (TransGen Biotech) according to the manufacturer's protocol then used for qualification through agarose gel electrophoresis and checked RNA integrity using the Agilent 2100.

### Sequencing

The RNA library was generated using NEBNext® Ultra™ RNA Library Prep Kit following manufacturer's recommendations. The mRNA was purified, and then used for cDNA synthesis and Illumina Hiseq platform sequencing.

### RNA-Seq Data Processing Pipeline

Clean reads were obtained by removing low quality reads and the adapter. The clean reads were aligned to *Toxoplasma gondii* TgME49 genome (ToxoDB-41) and pig genome Sscrofa 10.2 (GCA_000003025.4) through HISAT software (version 2.0.4) using a default parameter. Quantification of gene expression level through FPKM, differential expression analysis was performed through DESeq R package (version 1.10.1) with default parameter, then GO and KEGG enrichment analyses of differentially expressed genes were performed for functional prediction through GOSeq version 2.12 and KOBAS version 2.0, respectively. The GO term and KEGG pathways were sorted by corrected *p*-value then *p*-value.

### Real Time Quantitative RT-PCR (qRT-PCR)

To verify some of the RNA-seq data, the TB Green™ Premix Ex Taq ™ II (Takara) was used to quantify some of the genes of interest that are expressed differentially, according to the manufacture's protocol. All the primers used in this study were listed in Supplementary Table 5 in the supplemental materials section. Total RNA extracted from macrophages infected with *T. gondii* and control (uninfected) macrophages were reverse transcribed to single-stranded cDNA using the PrimeScript™ RT reagent Kit with gDNA Eraser (Takara). The real time quantitative qRT-PCR was performed in triplicate on the ViiA 7 Real-Time PCR System (Applied Biosystems) with the following conditions: 2 min at 50°C, 10 min at 95°C, 40 cycles of 95°C for 10 s, 60°C for 10 s and 72°C for 20 s. The comparative Ct (ΔΔCt) method was applied to determine the expression levels of the target gene relative to the housekeeping gene pig β-*actin* (Schmittgen and Livak, 2008).

## Data Availability Statement

The datasets generated for this study can be found in the Gene Expression Omnibus (GEO), accession No. GSE153330.

## Author Contributions

YZ, BS, and JZ conceived and designed the experiments. YS, LS, and XW performed the experiments, analyzed the data, and wrote the paper. MH and RF revised the manuscript. All authors read and approved the final version of the manuscript.

## Conflict of Interest

The authors declare that the research was conducted in the absence of any commercial or financial relationships that could be construed as a potential conflict of interest.
